# Progressive Medicine

**Published:** 1903-07

**Authors:** 


Progressive Medicine.—Fifth Annual Series. Volume I, March, 1903. A Quarterly Digest of Advances, Discoveries and Improvements in the Medical and Surgical Sciences. Edited by Hobart Amory Hare, M. D., Professor of Therapeutics and Materia Medica in the Jefferson Medical College of Philadelphia. Octavo, handsomely bound in cloth, 450 pages, illustrated. Per volume, $2.50, by express, prepaid. Per annum, in four cloth-bound volumes, $10.00. Lea Brothers & Co., Publishers, Philadelphia and New York.

In this number of "Progressive Medicine" Chas. H. Frazier reviews the wonderful progress which has lately been made in the surgery of the skull and brain. He also discusses the surgery of the neck and chest. Herrick, in his section on "Infectious Disease," dwells particularly upon the status of serum therapy at the present time. Crandall on the Diseases of Children, L. Hektoen on Pathology, Turner on Laryngology and Rhinology, and Randolph on Otology, all present interesting reviews of the work recently done in their respective lines. R. and L.

